# A longitudinal analysis of factors associated with age-related cataract among older Australian women: a cohort study of 7851 older Australian women 79–90 years

**DOI:** 10.1007/s11845-022-03130-7

**Published:** 2022-08-17

**Authors:** Mitiku Teshome Hambisa, Xenia Dolja-Gore, Julie E. Byles

**Affiliations:** 1 Centre for Women’s Health Research, University of Newcastle, Callaghan, NSW 2308 Australia; 2 Centre for Clinical Epidemiology and Biostatistics, University of Newcastle, Callaghan, NSW 2308 Australia; 3 School of Public Health, Haramaya University College of Health and Medical Sciences, P. O. Box 235, Harar, Ethiopia; 4 Neuroscience Research Australia, Sydney, NSW Australia; 5 School of Psychology, University of New South Wales, Sydney, NSW 2031 Australia

**Keywords:** Age-related cataracts, Increasing age, Older women, Systemic diseases

## Abstract

**Background:**

Age-related cataracts are a significant global health issue due to population ageing. More than 70% of older Australians aged 80 or above have clinically significant age-related cataracts.

**Aim:**

The study aimed to identify factors associated with age-related cataracts among older Australian women 79–90 years.

**Method:**

A 6-year longitudinal analysis of the Australian Longitudinal Study on Women’s Health (ALSWH) was conducted on 7117 women from surveys four to six. The women were asked whether they had been diagnosed or treated for cataracts 3 years before each survey. We used generalised estimating equation (GEE) modelling to identify factors independently associated with age-related cataracts.

**Results:**

At baseline (79–84 years), 44.8% lived in metropolitan Australia, 67.9% had good general health, 26.5% had private health insurance, 30.6% had cataracts, 28.8% had undergone cataract surgery, 12.0% had diabetes, 24.9% had skin cancer, 56.2% had hypertension, 24.0% had a history of falls, 63.0% had visited general practitioner (GP) frequently, and 48.8% were driving themselves as their main means of transport. In the final model, poor general health [adjusted odds ratio (AOR) = 1.23, 95% CI = 1.14, 1.33)], not driving (AOR = 1.09, 95% CI = 1.01, 1.18), having private health insurance (AOR = 1.13, 95% CI = 1.04, 1.23), frequent GP visits (AOR = 1.16, 95% CI = 1.07, 1.25), skin cancer (AOR = 1.26, 95% CI = 1.16, 1.37), hypertension (AOR = 1.13, 95% CI = 1.05, 1.21), and fall (AOR = 1.12, 95% CI = 1.04, 1.22) were significantly associated with the age-related cataracts.

**Conclusions:**

Systemic diseases, poor quality of life**,** driving cessation, and health service use were significantly associated with age-related cataracts in older women.

## Introduction

A cataract is the leading cause of blindness, responsible for approximately 51% of cases [1] and with 15·2 million cases among people aged 50 years and older in 2020 [2]. It is the preventable cause of blindness affecting 95 million people in the world [2, 3]. Cataract risk factors have hereditary and environmental components where the genetic factor plays a role in up to 70% of cases [4]. Age-related cataracts are the most common form of cataracts, generally occurring after 50 years [5]. The prevalence of cataracts increases with age, from around 4% among those aged 55–64 years to more than 90% among those aged 80 years and above [3]. As the population ages, the burden of cataracts will increase [6]. It is expected that age-related cataracts will continue to be an important global health issue due to increasing life expectancy [7], particularly among older women presenting huge financial burdens for both governments and individuals [6, 8]. At the age of 80, everyone either had a cataract or had undergone surgery [9, 10].

In Australia, cataracts are a common public health problem as a result of population ageing [11]. It is the second most common cause of bilateral vision impairment [11]. Clinically significant cataract was predicted to increase from 1.7 million people in 2001 to 2.7 million by 2021 in Australia [12]. Over 70% of older Australians aged 80 or above have clinically significant age-related cataracts, and the disease is more common among women than men [13]. The number of Australians with cataracts has grown by two-thirds during the past 20 years, indicating the impact of continued population ageing [12]. In 2016–2017, more than 700,000 Australians (3.9% of the whole population) were affected by cataracts [14]. According to the Medibank Better Health Index, there has been an increase in cataract incidence with 139,000 more Australians living with cataracts than in 2016–2017 compared to 2010–2011, with a steeper increase amongst older Australian women as the result of women’s greater longevity [14].

Previous studies have identified a number of factors associated with cataracts, including age [15], education [15], employment, socioeconomic factors, rural residence, smoking [15], excessive sunlight exposure [6, 16], diabetes [6, 15], and other systemic diseases [17].

Different studies examined smoking as a risk factor for cataracts, finding a consistently strong association [18–20]. Also, it has even been shown that there is a dose–response relationship between smoking and cataract that smoking cessation can decrease the risk of cataract [19]. Generally, smoking is one of the most constantly documented modifiable risk factors for cataract progression [6].

Diabetes has long been associated with cataracts, with evidence from different study designs and case observations [6, 15, 20, 21]. In addition, the high prevalence and early onset of cataracts and lens opacities in diabetic patients were also long-recognised by clinical studies [20, 22]. It has been suggested that around 4% of all cataracts can be attributed to diabetes [23].

An association of high prevalence of cataracts with hypertension was reported from the Barbados Eye Study [17] and a meta-analysis conducted on 25 studies in 2014. The pooled results showed that cataract risk in populations with hypertension significantly increased and concluded hypertension was strongly associated with cataract [24].

Many previous studies reported strong evidence for an increased association between falls and cataracts [25–28]. Fall is a public health problem and the main cause of an accident in older people as a result of frailty [29]. Even though cataract surgery is able to restore eyesight, lengthy waiting times for public patients, particularly those who have no private health insurance [30, 31] in many high-income countries, including Australia, is a common problem that results in falls while waiting for surgery [32, 33].

Several studies have reported driving cessation, reduced amount of driving, or decision to stop driving as a result of poor vision, often secondary to cataracts in old age [34, 35]. Driving depends heavily on vision. It is an almost well-established fact that eyesight is a very important organ required for driving activity [36]. Other research findings also revealed that eyesight problems, one of which is cataract, are among many commonly cited health issues mentioned as reasons for driving avoidance or cessation [35, 37].

In Australia, driving is a way of life and is common in older age groups [38]. The number of older road users Australians with a driver’s licence has been increasing at a rate greater than any other age group with a 44% increase for 65 years and above compared to what was only 17% growth across all age groups in 2014 [39]. This study hypothesizes as many Australians are driving across their life course; an age-related cataract will occur and may become a barrier in their way to continue driving at an advanced age.

In summary, previous studies identified sociodemographic factors like age, sex, education; lifestyle choices and health behaviours like smoking, alcohol drinking, obesity (BMI), and chronic conditions and injuries like diabetes, cardiac problem, hypertension, eye injury (surgery), falls, and UV-B exposure as factors associated with cataract. In turn, cataract has been associated with cessation or reduction in driving in older age.

However, what is less known is whether the same sociodemographic, lifestyle choices and chronic conditions are associated with age-related cataract at very old age and which factors are more important in relation to an ageing society and healthy ageing goals in older women. These factors have not been well explored in the very old age (80+) group. Previous studies of factors associated with cataracts have focused on middle-aged (40–54) and young-old (60–74) study populations. In this study, we question factors which are important in young, old and middle-aged people are the same for people over the age of 80 years [40, 41].

In addition, this study investigates the association between cataracts and driving in later life. Identifying the potential risk factors of age-related cataracts in this particular age group may help in targeting clinical interventions, early diagnosis, and prompt treatment that, in turn, reduces the economic, social and personal impact of cataract by improving the quality of life of older people in keeping with the goals of healthy ageing [42].

The specific objective of this study is to investigate factors associated with age-related cataracts among older Australian women from the age of 79–90 years. It is 6 years of follow-up using generalised estimating equation modelling (GEE) on the Australian Longitudinal Study on Women’s Health (ALSWH) data survey waves four to six.

## Methods section

### Study sample

The data for this study were from the 1921 to 1926 cohort of Australian Longitudinal Study on Women’s Health (ALSWH). ALSWH was established to examine the continuing health, medical condition, and health service utilisation of Australian women (www.alswh.org.au) [43]. The women were randomly sampled from the Australian Medicare database which includes all Australian citizens and permanent residents. They were first surveyed in 1996 when the women in the 1921–1926 cohort were 70–75 years. Since that time, the women have been surveyed every 3 years from 1996 to 2011, and then 6-monthly [43, 44]. This study uses survey four as a baseline when the cataract and eye surgery (including cataract) questions were incorporated into the ALSWH survey and include survey data from survey 4 (ages 79–84) to survey 6 (85–90) for longitudinal analysis.

In every survey, demographic factors; health and medical conditions including eye health, private health insurance, GP visit; well-being like general health; chronic medical conditions like skin cancer, diabetes, hypertension, and fall; environmental factors like state of residence (area); and lifestyle choices were inquired across the women’s life.

### Outcome variable

#### Cataract (self-reported diagnosis for cataract)

To identify having a cataract we used the survey question, “In the last 3 years have you been diagnosed with or treated for Cataract?” with the response option “Yes” and” No”. This question was asked across all three surveys.

#### Independent variables

Independent variables in this study were chosen based on previous literature [6, 45] and modelled according to the sociodemographic factors, health behaviours, and chronic medical conditions. All independent variable measures were taken from ALSWH surveys 4 to 6, except education, country of birth, hormone replacement therapy (measured at survey 1 and 3), language spoken at home which were only measured at survey 1 in 1996, and whether the women eat fruit or vegetables most days and smoking status measured at survey 2, skin surgery due to cancer or sunspots removal (at measured at surveys 2 and 3) and different types of medication use measured at survey 3, and alcohol use status measured at survey 3 and 6.

Sociodemographic factors included age, highest educational qualification, state, country of birth, short-listed categorisation of language spoken at home, and area of residence.

Health behaviour variables included were self-rated health, which was dichotomized as good (excellent/very good/good) versus poor (fair/poor), having private health insurance, and frequency of general practitioner (GP) visits in the last 12 months of the survey. In addition, whether the women eat fruit or vegetables most days, smoking status, whether they drive a car themselves or not (means of transport), and alcohol use status were treated as health behaviour variables.

Chronic medical conditions and injury that could affect cataract development and progression included skin cancer, diabetes, hypertension, stroke, hormone replacement therapy, different types of medication use and fall.

### Analysis

Data sets for surveys 4, 5, and 6 were merged and response patterns for the outcome variable (cataract) was performed to examine patterns of missing data. Independent variables were examined for correlation with each other, and bivariate association were assessed using chi-square test for categorical variables and *t*-tests for continuous variables with the outcome cataract.

The following variables, ARIA+, country of birth, education (also correlated with private insurance), language spoken at home, marital status, eating adequate amounts of fruit or vegetables most of the days, BMI, smoking, alcohol, were not statistically significantly associated with cataract on bivariate pre-regression screening (even though some of these variables seems to be well established risk factors in previous literature) and were excluded from the modelling process. Use of multiple medications was correlated with many variables including BMI, GP visit, and driving; skin surgery due to cancer or sunspots removal was correlated with skin cancer; physical function (PF) score and social function (SF) score were correlated with general health, GP visits and falls and were subsequently excluded from the modelling.

Generalised estimating equations (GEE) models were performed to account for the repeated measures of cataract over the three time points (6-year follow-up), and to adjust for multivariable relationships. Models were explored using time-invariant and time-varying independent variables, based on the Liang and Zeger approach [46]. GEE models were built applying binary distribution of the outcome variable, with a logit link function and autoregressive correlation structure as cataract was measured at equal time interval with 3 years between each survey from 2005 to 2011. Only independent variables with *p* < 0.25 were considered for the analyses using generalised estimating equations (GEE) model.

Four GEE models were constructed: model (1) a base model including time and baseline age. Driving was also added to examine the association between cataracts and driving, prior to adjusting for other variables; model (2) model 1 plus general health, smoking, GP use, and health insurance; model (3) model 2 plus health conditions. Lastly, in model 4, we looked at significant variables from model 3.

## Results

### Sample characteristics at baseline

The original cohort includes 12,432 women who joined the study in 1996. The response rate at this time was around 40%, but the women were broadly representative of the general population of women 70–75 in comparison with national data [47]. Survey 4 was completed by 7158 women, and 2289 women had died by that time. A further 19 women had no information for cataract for surveys 4–6, and 122 women had missing data for the outcome variable at baseline. At this point, 7117 women remained in the sample for subsequent longitudinal analysis.

Table 1 shows sociodemographic and health-related characteristics of the participants at baseline. Around two-thirds (63.1%) were non-partnered, 39.7% had school certificate, 13.2% had high school certificate, and 17.1% had trade/college or university degree, 44.8% live in metropolitan, 36.72% live in inner regional, 18.5% live in outer regional, remote, and very remote Australia, and 48.8% were driving themselves as their main means of transport.

**Table 1:** Baseline characteristics of study participants according to cataract status, 2021

**Baseline explanatory variables**	**Total**	**Participants according to cataract status (*N* = 7117)**		***P*-value**
		**Yes** **2180 (30.63%)**	**No** **4937 (69.37%)**	
**Sociodemographic factors**				
**Marital status**				0.25
Partnered	2612 (36.93)	743 (34.3)	1869 (38.1)	
Non-partnered	4461 (63.07)	1423 (65.7)	3038 (61.9)	
**Age in years (mean, SD)**	80.79 (1.43)			<.0001
**Educational qualification**				0.5503
No formal qualifications	2040 (30.03)	632 (30.6)	1408 (29.8)	
School certificate	2699 (39.73)	833 (40.3)	1866 (39.5)	
High School Certificate	894 (13.16)	256 (12.4)	638 (13.5)	
Trade/college or university	1161 (17.09)	346 (16.7)	815 (17.2)	
**Driving**				0.0038
Yes	3271 (48.79)	945 (46.1)	2326 (49.9)	
No	3433 (51.21)	1104 (53.9)	2329 (50.1)	
**Country of birth**				0.6789
Australian born	5351 (79.62)	1637 (79.2)	3714 (79.8)	
Other English-speaking country	846 (12.59)	259 (12.6)	587 (12.6)	
Europe/Asia/other	524 (7.80)	170 (8.2)	354 (7.6)	
**Accessibility/remoteness index for Australia (ARIA +)**				0.8095
Major cities	3186 (44.78)	965 (44.3)	2221 (45.0)	
Inner regional	2613 (36.73)	812 (37.3)	1801 (36.5)	
Outer regional/remote/very remote	1316 (18.50)	402 (18.4)	914 (18.5)	
**Health behaviour and lifestyle variables**				
Private health insurance**				0.0175
Yes	1864 (26.48)	611 (28.2)	1253 (25.7)	
No	5175 (73.52)	1553 (71.8)	3622 (74.3)	
**General health**				<.0001
Good	4549 (67.87)	1335 (62.8)	3214 (70.2)	
Poor	2154 (32.13)	791 (37.2)	1363 (29.8)	
**GP visit**				<.0001
4 or less	2581 (36.98)	678 (36.7)	1903 (39.3)	
5 or more	4398 (63.02)	1463 (68.3)	2935 (60.7)	
**Alcohol status**				0.3194
Drinker	2621 (39.01)	786 (38.1)	1835 (39.4)	
Non-drinker	4098 (60.99)	1276 (61.9)	2822 (60.664)	
**Smoking status**				0.142
Never-smoker	4250 (65.75)	1293 (64.5)	2957 (66.3)	
Ex-smoker/current smoker	1950 (30.17)	713 (35.5)	1501 (33.7)	
**Chronic conditions**				
Diabetes **				0.0009
Yes	847 (11.98)	304 (13.9)	543 (11.1)	
No	6225 (88.02)	1876 (86.1)	4349 (88.9)	
**Skin cancer**				<.0001
Yes	1759 (24.87)	617 (28.3)	1142 (23.3)	
No	5313 (75.13)	1563 (71.7)	3750 (76.7)	
**Hormone replacement therapy**				0.0105
Yes	5828 (89.66)	1769 (88.2)	4059 (90.3)	
No	672 (10.34)	237 (11.8)	435 (9.7)	
**Fall**				0.0036
Yes	1647 (23.97)	552 (26.2)	1095 (23.0)	
No	5225 (76.03)	1552 (73.8)	3673 (77.0)	
Hypertension**				<.0001
Yes	3992 (56.23)	1307 (60.0)	2685 (54.6)	
No	3107 (43.77)	873 (40.0)	2234 (45.4)	
Stroke**				0.0069
Yes	282 (3.99)	108(5.0)	174 (3.6)	
No	6790 (96.01)	2072(95.0)	4718 (96.4)	
**Cataract surgery**				<.0001
Yes	1851 (28.8)	1493 (71.1)	358 (8.3)	
No	4568 (71.2)	607 (28.9)	3961 (91.7)	
Other eye surgeries**				<.0001
Yes	334 (5.20)	149 (7.1)	185 (4.3)	
No	6085 (94.80)	1951 (92.9)	4134 (95.7)	

Regarding health-related characteristics, 30.6% had cataract, 28.8% had underwent cataract surgery, 67.9% had excellent, very good or good general health, 26.5% had private health insurance, 12.0% had diabetes, 24.9% had skin cancer, 56.2% had hypertension, 24.0% had a history of fall, and 63.0% had visited GP 5 or more times in the past 12 months of the study (Table 1).

**Fig. 1:**
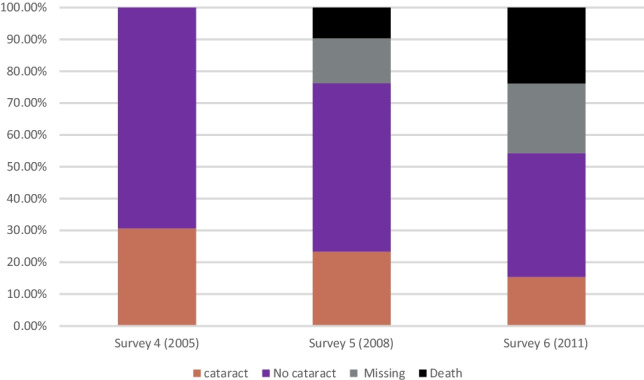
Proportion of women having cataract from 79 to 90 years surveys 4–6 (2005–2011)

Three years later, at survey 5 (age 82–87 years), 23.3% of the women had cataracts, some deceased, and some did not complete the survey (Fig. 1, progression chart). A smaller proportion, 15.4%, had cataracts at survey six at age 85–90 years, with many women deceased or otherwise did not respond to the survey. However, among respondents, the prevalence of cataract remained at around 30% at each survey: 30.6% at survey four, 31.2% at survey five and 28.1% at survey six. It should be also noted that some of these women had undergone cataract surgery. For instance, at baseline (ages 79–84 years), 28.8% had already undergone cataract surgery (Table 1).

### Factors associated with age-related cataracts

Area of residence, alcohol consumption, and education have no association at bivariate screening. Time was significant between baseline and survey 6 but not with survey 5, and little change with addition of other variables.

Baseline age was not significant across the modelling process. However, we kept age in the model regardless of its statistical significance, as age is very important risk factor for cataracts.

Not driving was significantly associated with having cataracts when added to baseline age. As the model building continues, not driving was significantly associated with having cataracts with little change in effect when lifestyle choice, health behaviour, and chronic condition variables added to the model across model 2 to model 4 (AOR = 1.09, 95% CI = 1.01, 1.18) (Table 2, model 4). From the lifestyle and health behaviour variables, smoking was not significantly associated with cataract (Table 2, model 2). From chronic conditions, diabetes, stroke, other eye surgeries, and hormone replacement therapy were not significant (Table 2, model 3).

**Table 2:** Factors associated with cataract among older Australian women (age 79–90 years) over time, 2021

**Explanatory variables**	**Model 1** **(sociodemographic factors)**	**Model 2** **(health behaviours and lifestyle)**	**Model 3** **(chronic conditions)**	**Model 4** **(nested, fully adjusted model)**
	**Adjusted odds ratio (95% confidence intervals)**			
**Intercept**	1.02 (0.13, 8.15)	1.83 (0.20, 16.3)	0.84 (0.09, 7.72)	0.94 (0.08, 10.4)
**Time** Survey 4 (2005, age 79–84 years) (ref)	1.0	1.0	1.0	1.0
Survey 5 (2008, age 82–87 years)	1.06 (0.96, 1.8)	1.06 (0.95, 1.18)	1.04 (0.93, 1.16)	0.95 (0.87, 1.02)
Survey 6 (2011, age 85–90 years)	0.95 (0.80, 1.14)	1.003 (0.83, 1.21)	0.95 (0.79, 1.15)	0.78 (0.71, 0.87)
**Sociodemographic factors**				
**Age in years ≠ **	0.99 (0.96, 1.01)	0.98 (0.95, 1.01)	0.99 (0.96, 1.02)	0.99 (0.96, 1.01)
**Driving**				
Yes	1.0	1.0	1.0	1.0
No	1.11 (1.03, 1.20) *	1.08 (1.01, 1.18) *	1.09 (1.01, 1.19) *	1.09 (1.01, 1.18) *
**Health behaviours and lifestyle choices**				
General health*				
Good		1.0	1.0	1.0
Poor		1.28 (1.18, 1.38) *	1.24 (1.15, 1.35) *	1.24 (1.13, 1.35) *
**Smoking status**				
Never-smoker		1.0		
Ex-smoker/current smoker		1.08 (0.98, 1.17)		
GP visits*				
4 or less		1.0	1.0	1.0
5 or more		1.19 (1.10, 1.29) *	1.12 (1.03, 1.22) *	1.11 (1.02, 1.21) *
Private health insurance*				
Yes		1.16 (1.07, 1.27) *	1.12 (1.03, 1.22) *	1.16 (1.06, 1.27) *
No		1.0	1.0	1.0
**Chronic conditions**				
**Diabetes**				
Yes			1.13 (0.99, 1.29)	
No			1.0	
Skin cancer*				
Yes			1.26 (1.16, 1.37) *	1.22 (1.12, 1.34) *
No			1.0	1.0
Hypertension*				
Yes			1.12 (1.03, 1.21) *	1.13 (1.04, 1.23) *
No			1.0	1.0
Fall*				
Yes			1.15 (1.05, 1.25) *	1.13 (1.04, 1.24) *
No			1.0	1.0
**Hormone replacement therapy**				
Yes			0.92 (0.81, 1.04)	
No			1.0	
**Stroke**				
Yes			1.11 (0.93, 1.32)	
No			1.0	
**Other eye surgeries**				
Yes			1.16 (0.97, 1.39)	
No			1.0	

Key: *significant at p < 0.05, ≠ “age” was adjusted and kept across the model regardless of its non-significant association

In the final multivariable nested model, poor general health (AOR = 1.23, 95% CI = 1.14, 1.33), having private health insurance (AOR = 1.13, 95% CI = 1.04, 1.23), 5 or more GP visits in the past 12 months prior to the survey (AOR = 1.16, 95% CI = 1.07, 1.25), skin cancer (AOR = 1.26, 95% CI = 1.16, 1.37), hypertension (AOR = 1.13, 95% CI = 1.05, 1.21), and fall (AOR = 1.12, 95% CI = 1.04, 1.22) were significantly associated with the odds of having age-related cataracts (Table 2, model 4). The odd ratios indicated in this document is that of the parsimonious adjusted nested model 4.

## Discussion

In this population-based longitudinal study of older Australian women, we found that poor general health, not driving, having private health insurance, frequent GP visits from health behaviour and lifestyle factors were significantly associated with age-related cataracts. There was also a strong significant association between hypertension, skin cancer, and fall and age-related cataracts from chronic conditions. In this study, previously reported associations between hypertension, driving cessation, fall and cataracts were confirmed. However, there was no association between smoking, alcohol intake, diabetes, hormone replacement therapy, and age-related cataracts in the current study.

### Health behaviour, lifestyle choices, and age-related cataracts

In many studies, poor self-rated health has been associated with visual impairment, which is usually due to cataracts in older people [48, 49]. Our study also confirmed that women who rated their general health as poor were 23% more likely to have cataracts (Table 2 model 4). This might be due to its effect of cataract-related vision impairment on mental health, driving, social interaction, risk of falling and then fracture. As evidenced in previous studies, driving cessation due to cataract-related vision problems may affect older people’s independence, freedom of movement to fulfil their basic needs, essential services like shopping groceries and medical supplies [50–52]. It may also affect their mental health [53], risk of falls and then fracture, which hampers the quality of life in older individuals [25]. In general, this can be seen as indicative of the poor quality of life in older persons as a result of cataracts or change in vision and its consequences if timely intervention is not done [32, 54]. This finding has critical importance as the evaluation of healthcare in older people mainly focuses on quality of life.

In this study, women who were not driving were 9% more likely to have cataracts by the age of 79–90 years (Table 2 model 4). Numerous previous researchers reported driving cessation or decision to stop driving was mainly due to vision impairment as a result of cataracts in older age [34, 50, 52, 55]. Our study also confirmed this fact which is in line with many study findings as this could be due to the fact that driving a motor vehicle was strongly depends on the visual function of the eye above any other sense organs as receiving and processing information to operate a motor vehicle when driving is mainly received through eye [35, 36, 50, 56].

It is an almost well-established fact that eyesight is a very important organ required for driving activity [36]. Other research findings also revealed eyesight problems, one of which is due to cataracts, are among many commonly cited health issues mentioned as reasons for driving avoidance or cessation [35, 37]. A cataract is the main public health problem in older Australians; as well above 70% of older Australians had cataracts by the age of 80 years, and the problem is increasing with age and more common in women than men. It can affect the mental, physical, social-wellbeing, and more importantly, the functional ability of older people, which has paramount importance for healthy ageing [13].

This finding is very important as the number of older road user Australians have been increasing as a result of population ageing [39], as well as many Australians are driving across their life course. But age-related eye conditions, like cataracts, will occur in their way and become a barrier to driving which impacts older women not continuing to drive in old age, which needs early identification of risk factors, prioritizsing, and targeted intervention to promote healthy ageing of older Australians. The present study revealed that those women who have private health insurance were 23% more likely to be diagnosed or treated for cataracts compared to those who did not have private insurance, and those women with frequent GP (general practitioner) visits were also 16% more likely to be diagnosed or get treatment for cataract (Table 2 model 4). Recently published large population-based study of 257,237 participants of Australian population from the 45 and Up Study, having private health coverage, was independently associated with the treatment of cataract privately [57]. In another study in 4 European Countries, namely Germany, France, Italy, and Spain, having private health insurance coverage provided French patients with more and better access to the more effective and sophisticated eye lens [31].

The association between frequent GP use and cataract in the current study could be explained by the fact that when patients visit a family doctor or their own GP or health care intuition like a hospital for other comorbidity or ocular disease, they may get the opportunity to be diagnosed and referred early. A study conducted in Sweden also revealed this fact where more than 41% of patients who regularly visit their GP were diagnosed and referred by health care providers they frequently met compared to those who initiated and sought treatment by themselves [58].

Therefore, our findings and the limited previous studies suggest the importance of having private insurance as equity and accessibility to health services. The frequent GP visits also might show availability and accessibility of healthcare facilities, good health-seeking behaviour, or comorbidities needing frequent healthcare provider visits, especially in older women, which may also suggest socioeconomic inequalities. This could suggest the need for targeted intervention for those who have no private health cover in addition to Medicare cover.

### Systemic diseases and chronic conditions associated with age-related cataracts

In our study, hypertension was associated with cataracts, where those women with hypertension were 1.13 times more likely to have cataracts compared to those without hypertension (Table 2 model 4) in the final adjusted model after controlling several confounding factors. In Australia’s ‘Blue Mountains Eye Study’, it was reported repeatedly that hypertension and other cardiovascular risk factors were associated with an incident of different forms of age-related cataract [59, 60] and many others studies, including a meta-analysis conducted on 25 studies in 2014 where the pooled results revealed that cataract risk in populations with hypertension was significantly increased, but in cataract subtype analysis hypertension has no association with nuclear cataract [24]. Finally, the study concluded that hypertension is associated with cataract development which might be either because of hypertension or anti-hypertension drugs utilisation [24, 60]. It also might be due to alteration of protein structure in the lens capsules, as suggested by Lee et al. [61]. In another study, the Barbados Eye Study, the association of high occurrence of cataracts with hypertension was reported [17]. Our study also revealed the association between hypertension and cataract, but the pathophysiology behind its formation and progression is unclear even though several suggestions have been forwarded [24]. Therefore, proper control of hypertension among older people is important, and further research is needed to identify the pathophysiology between hypertension and cataract in order to establish a causal pathway.

Only a few other studies have investigated the association between skin cancer and age-related cataracts. The current study has discovered that there was a strong positive association between skin cancer and cataract in which the odds of developing cataracts were 1.26 times more likely in older women with skin cancer compared to those who had no skin cancer (Table 2 model 4). This finding is relatively new of its kind as only two cross-sectional studies so far reported the positive association between skin cancer and age-related cataract as to the researchers’ knowledge [62, 63]. One of the studies was conducted on older Australian populations using historical Cohort of the Registry of Senior Australians (ROSA) that reported age-related cataract was positively associated with skin cancer and its different types among people aged ≥ 65 years (≥ 50 years in case of Aboriginal or Torres Strait Islander descent) who had an aged care eligibility assessment between 2005 and 2015 [63]. The other cross-sectional study conducted in Israel also examined the association of skin cancer with cataracts using a local health care services database among persons 40 years and over in which positive association between cancer skin lesion (cancer) from exposure to sunlight and cataract was identified [62]. Our study also revealed the same fact with a more sophisticated population-based longitudinal analysis among an older cohort of Australian women from age 79–84 to 85–90, which strengthened the previous studies.

The finding may be explained as exposure to sunlight may exacerbate the development and progression of age-related cataracts as ultraviolet radiation from sunlight exposure is the established environmental risk factor for both skin cancer and cataract [6, 16, 62, 64]. This effect was also demonstrated in the study of the Australian population that revealed a high level of climatic ultraviolet radiation was associated with the increasing prevalence of cataracts among indigenous Australians [65]. The finding supports the hypothesis previously suggested that both skin cancer and age-related cataract may share common causal pathways like ultraviolet radiation, particularly from sunlight exposure [63]. This finding can also be used as a proxy indicator of ultraviolet radiation from sunlight exposure as a risk factor for cataracts, as we do not have ultraviolet radiation (sunlight exposure) variable in our data, which would be advantageous to address the limitation of this study. This study can be used by clinicians to consider and manage multiple diseases (multimorbidity) such as ocular, dermatologic, and systemic diseases when patients visit for one health problem for early identification and referral. Future studies should focus on identifying the common causal pathway between skin cancer and cataract, testing the suggested hypothesis that cataracts and skin cancer share common pathophysiology, given that ultraviolet radiation from sunlight exposure is a common risk factor.

In our study, the odds of falls were 13% more likely associated with having cataracts among older women compared to those without a history of falls (Table 2 model 4). Similar to our finding, many previous studies also reported a significant association between falls and cataracts among older people in different settings, including Australia [30, 31]. As age increases, the risk of falls also considerably increases, and it becomes a public health problem [40, 73], resulting in fractures and other injury [66, 67], hospitalizations [68, 69], admission to aged care [68], and decline in quality of life, particularly in older people with cataracts [32, 68, 70, 71]. In the Melbourne visual impairment study, researchers have identified the risk of falls in older people with cataracts was almost three times more than for those without cataract [72]. In 2015–2016 alone, the treatment, functional loss, disability, and deaths due to falls were estimated to cost the Australian healthcare system $3.9 billion [73].

Against this cost of falls, cataract surgery is a high impact procedure that restores sight. However, lengthy waiting times for public patients, particularly those who have no private health insurance in many high-income countries such as Australia, are common. This supply side problem in accessing care can exacerbate the risks of falls while waiting for surgery [32, 33].

In Australia, cataract surgery waiting time can take up to 3 years from diagnosis to treatment, with 2 years spent in referral and pre-treatment ophthalmologic screening [74], and about 12 months on a surgical waiting list [75]. For instance, 90% of patients waited for 334 days for surgery in 2017–2018, and about 2.5% of these patients waited for over a year [75]. However, a randomised controlled trial study conducted in the UK by Harwood et al. [76] revealed decreasing the cataract surgery waiting times from 1 year to 1 month has resulted in a decrease in the rate of falls by 34% [76], revealing many cases of falls in older people with cataract can be prevented. Therefore, the issue of cataract surgery is not only just surgery but also the issue of preventing falls and fractures, promoting driving (mobility) and independence, and reducing admission to hospital and residential aged care. Thus, early cataract treatment by reducing surgical waiting time can help prevent falls and fractures and can promote healthy ageing. Training country tailored mid-level health practitioners like cataract surgeons may help to reduce the burden on ophthalmologists, rather than having patients waiting for specialist service all the time, specifically for those who have no private health insurance. This will help to ensure the right to sight and improve the quality of life.

This study has some limitations. Firstly, the cataracts and other variables are self-reported. This is a common limitation for large epidemiological surveys. We also do not have data for excessive sunlight (ultraviolet radiation) exposure from sunlight which is the established risk factor for age-related cataracts in several previous studies. But in our study, we identified a strong association between skin cancer and cataract, and we believe we addressed this through the association between skin cancer and cataract as ultraviolet sunlight exposure is a common risk factor for both skin cancer and cataract. In addition, we did not identify the three different subtypes of cataracts in this study, and it should be noted that the finding is for all three types of cataracts.

## Conclusion

This finding supports our hypothesis that vision impairment due to age-related cataracts may be the barrier to continue driving in old age. The finding also supports our hypothesis that factors associated with age-related cataracts in young-old and middle-aged people may not be the same for people aged 80 years and above as demographic characteristics and lifestyle choices are different, and there have been competing risks over the course of their later lives. In our study, chronic conditions such as hypertension, skin cancer, and falls and health behaviours such as frequent GP visits, poor general health, and private health insurance were significantly associated with age-related cataracts in late life. However, sociodemographic and lifestyle factors like education, smoking, and alcohol intake were not associated with age-related cataracts in this age group. However, they have been shown to have an association in young-old and middle-aged individuals.

Policymakers should consider specific and targeted policy and practice for this oldest age group that addresses access to health service use like encouraging optimum GP visits, prioritising those with no private insurance, and reducing cataract surgery waiting times.

Also, integrating eye health into non-communicable chronic diseases will have importance. The findings can be used by policymakers and clinicians to consider managing multiple diseases (multimorbidity) such as ocular, dermatologic, and systemic diseases when patients visit for one health problem. This has paramount importance for early identification and referral in relation to an ageing society and healthy ageing goals.
